# ATP‐independent molecular chaperone activity generated under reducing conditions

**DOI:** 10.1002/pro.4378

**Published:** 2022-07-13

**Authors:** Axel Leppert, Gefei Chen, Danai Lianoudaki, Chloe Williams, Xueying Zhong, Jonathan D. Gilthorpe, Michael Landreh, Jan Johansson

**Affiliations:** ^1^ Department of Biosciences and Nutrition Karolinska Institutet Huddinge Sweden; ^2^ Department of Microbiology, Tumour and Cell Biology Karolinska Institutet Solna Sweden; ^3^ Department of Integrative Medical Biology Umeå University Umeå Sweden; ^4^ Division of Structural Biotechnology, Department of Biomedical Engineering and Health Systems, School of Engineering Sciences in Chemistry, Biotechnology and Health (CBH) KTH Royal Institute of Technology Huddinge Sweden

**Keywords:** ATP‐independent molecular chaperone, Bri2 BRICHOS, BRICHOS domain, disulfide bond formation, extracellular protein aggregation

## Abstract

Molecular chaperones are essential to maintain proteostasis. While the functions of intracellular molecular chaperones that oversee protein synthesis, folding and aggregation, are established, those specialized to work in the extracellular environment are less understood. Extracellular proteins reside in a considerably more oxidizing milieu than cytoplasmic proteins and are stabilized by abundant disulfide bonds. Hence, extracellular proteins are potentially destabilized and sensitive to aggregation under reducing conditions. We combine biochemical and mass spectrometry experiments and elucidate that the molecular chaperone functions of the extracellular protein domain Bri2 BRICHOS only appear under reducing conditions, through the assembly of monomers into large polydisperse oligomers by an intra‐ to intermolecular disulfide bond relay mechanism. Chaperone‐active assemblies of the Bri2 BRICHOS domain are efficiently generated by physiological thiol‐containing compounds and proteins, and appear in parallel with reduction‐induced aggregation of extracellular proteins. Our results give insights into how potent chaperone activity can be generated from inactive precursors under conditions that are destabilizing to most extracellular proteins and thereby support protein stability/folding in the extracellular space.

**Significance:**

Chaperones are essential to cells as they counteract toxic consequences of protein misfolding particularly under stress conditions. Our work describes a novel activation mechanism of an extracellular molecular chaperone domain, called Bri2 BRICHOS. This mechanism is based on reducing conditions that initiate small subunits to assemble into large oligomers via a disulfide relay mechanism. Activated Bri2 BRICHOS inhibits reduction‐induced aggregation of extracellular proteins and could be a means to boost proteostasis in the extracellular environment upon reductive stress.

## 1 INTRODUCTION

In order to maintain proteostasis, cells invest in a large integrated network of structurally and functionally diverse molecular chaperones.^1^ One important class are energy (ATP)‐independent molecular chaperones that have the ability to increase solubility of un‐ or misfolded intermediates and thereby increase cell‐stress resistance.^2^ Considering that the ATP concentration in the extracellular environment is several fold lower compared to that in the cytosol, ATP‐independent molecular chaperones likely play an important role in regulating extracellular proteostasis.^3^ However, much of what is known about the functions of ATP‐independent molecular chaperones relate to intracellular proteins, such as the small heat shock proteins (sHSPs) and only few extracellular chaperones have been identified. This is surprising considering that extracellular deposits of aggregated protein are found in a variety of human diseases.^4^

The Bri2 BRICHOS domain is the extracellularly released region of the Bri2 protein, which is a type II transmembrane protein that undergoes several proteolytic processing steps along the secretory pathway.^5^, ^6^, ^7^ In isolation, the Bri2 BRICHOS domain exists in different assembly states of which monomers and dimers are very potent in preventing fibrillation and associated toxicity of the Alzheimer disease related amyloid‐β peptide.^8^, ^9^ In contrast to monomers and dimers, polydisperse high‐molecular‐weight (HMW) Bri2 BRICHOS assemblies are the only species that reduce non‐fibrillar protein aggregation similar to classical ATP‐independent sHSPs, like αB‐crystallin or HSP27.^8^, ^10^ However, the underlying mechanism and conditions that lead to the formation of HMW Bri2 BRICHOS assemblies have remained elusive.

There are two strictly conserved cysteine residues in the BRICHOS domain^11^, ^12^ that form an intramolecular disulfide bridge in the monomer, while in HMW oligomers, the subunits are largely cross‐linked through intermolecular disulfide bonds.^8^ The redox status is essential for the correct folding of proteins and reversible reduction, oxidation, and disulfide bond formation of cysteine residues provide a handle to control a broad range of different protein conformations and functions.^13^ Here, we show that reducing conditions, which promote disulfide‐rich extracellular proteins to aggregate, also promote the Bri2 BRICHOS domain to assemble into an efficient ATP‐independent molecular chaperone, through a distinct disulfide bond reshuffling relay. This mechanism enables the Bri2 BRICHOS domain to respond to potentially harmful reducing conditions in an overall oxidizing environment, like the extracellular space.

## 2 RESULTS

### 2.1 Bri2 BRICHOS oligomer assembly is triggered under reducing conditions

**FIGURE 1:**
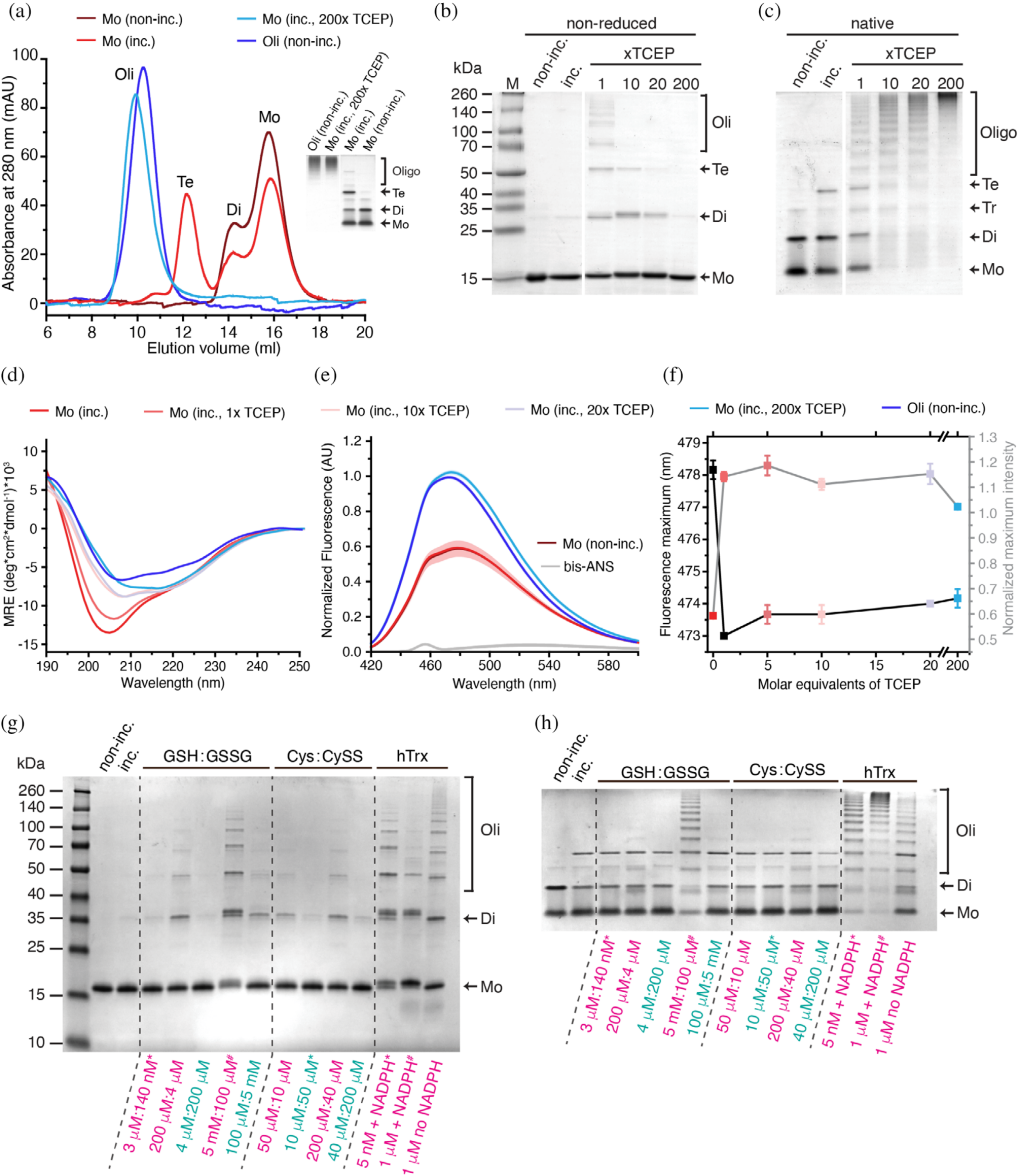
Reduction‐induced formation of Bri2 BRICHOS HMW assemblies. Bri2 BRICHOS samples are marked as: non‐incubated (non‐inc.) and incubated in the absence or in the presence of reductant (inc.). Assembly states are highlighted as Mo, monomer; Di, dimer; Te, tetramer; Oli, oligomer. (a) SEC elution profiles and native PAGE (inset) of monomers incubated in the absence and presence of TCEP, and non‐incubated oligomers isolated from *E.Coli*. (b) Non‐reducing SDS‐PAGE and (c) native PAGE analysis of Bri2 BRICHOS monomers incubated in the absence or presence of increasing molar ratios of TCEP. (d) Far‐UV CD spectra and (e) bis‐ANS fluorescence spectra of different Bri2 BRICHOS samples. The red and the brown curves in (e) overlap and fluorescence values have been normalized to non‐incubated Bri2 BRICHOS oligomers. (f) Changes in the bis‐ANS fluorescence maximum (left scale, black line) and the maximum fluorescence intensity normalized to non‐incubated Bri2 BRICHOS oligomers (right scale, grey line). All data are presented as mean values ± *SD* of 3 individual replicates. (g) Non‐reducing SDS‐PAGE and (h) native PAGE analysis of Bri2 BRICHOS monomers incubated with various redox buffer systems. Excess of the reducing equivalent is highlighted in magenta and excess of the oxidizing equivalent in green. Physiological redox couple concentrations (see Table S1) are indicated with # (intracellular) or * (extracellular).

To investigate reduction‐induced changes in the quaternary organization of the Bri2 BRICHOS domain, different Bri2 BRICHOS species, that is, monomers, dimers, and oligomers were expressed and purified from *E. coli*, as previously described.^8^ Under non‐reducing conditions, no free thiols in Bri2 BRICHOS monomer (−0.01 ± 0.07 thiols/mol), dimer (0.04 ± 0.01 thiols/mol), or oligomer (0.03 ± 0.02 thiols/mol) preparations were detected, showing that all cysteine residues are engaged in intra‐ and/or intermolecular disulfide bonds. Size exclusion chromatography (SEC) analysis reveals that non‐incubated monomers form an equilibrium between monomers and dimers, and during incubation at 37°C, a fraction of the molecules assembles into tetramers (Figure 1A). The association of monomers into dimers/tetramers occurs also in a concentration‐dependent manner (without incubation) reaching a plateau where ~34% of monomers are assembled into dimers and tetramers (Figure S1). SDS‐PAGE and native PAGE analyses show that these dimers and tetramers are largely non‐covalently linked. Using characteristic tryptophan fluorescence emission changes upon dimer formation of a Thr to Trp Bri2 BRICHOS mutant,^14^ an apparent K_D_ of 1.0 ± 0.2 μM was calculated for the assembly formation (Figure S1). However, adding a 200‐fold molar excess of the reductant TCEP converts monomers entirely into a polydisperse mixture of HMW assemblies, with a similar size compared to oligomers isolated from *E. coli* (Figure 1A). At an equimolar concentration, TCEP induces the formation of polydisperse disulfide‐dependent Bri2 BRICHOS assemblies and increasing the molar excess of TCEP shifts the equilibrium toward higher‐order oligomers (Figure 1B,C).

Far‐ultraviolet (UV) circular dichroism (CD) spectroscopy revealed that the overall secondary structure converts from a high content of random coils, as typically observed for Bri2 BRICHOS monomers, toward the more structured oligomer conformation in a TCEP concentration‐dependent manner (Figure 1D). To gain insights into the stability of Bri2 BRICHOS monomers and oligomers, we performed thermal denaturation experiments using UV‐CD spectroscopy (Figure S2). Under non‐reducing conditions, where all disulfide bonds are intact, Bri2 BRICHOS monomers showed some degree of reversible structural flexibility compared to the structurally rigid oligomer. Under reducing conditions, by the addition of a 200‐fold molar excess of TCEP, Bri2 BRICHOS monomers behave similar as under non‐reducing conditions during heating but adopt a secondary structure similar to non‐incubated oligomers after cooling. In contrast, Bri2 BRICHOS oligomers show only minor structural changes under reducing conditions, and the transition during heating is fully reversible.

Small HSPs are able to bind substrate proteins by hydrophobic interactions.^15^, ^16^ We used the extrinsic fluorescent dye 4,4′‐bis‐1‐anilinonaphthalene‐8‐sulfonate (bis‐ANS), to measure reduction induced changes of the exposed surface hydrophobicity of Bri2 BRICHOS proteins (Figure 1E). Non‐incubated and Bri2 BRICHOS monomers incubated in the absence of TCEP show near identical bis‐ANS fluorescence emission spectra. However, addition of TCEP leads to a shift of the fluorescence emission maximum toward shorter wavelengths and an increase of the maximum fluorescence intensity to values comparable to oligomers isolated from *E.coli*. These characteristic changes in the fluorescence emission spectra, indicative of an increase in the overall surface hydrophobicity, saturate at equimolar TCEP concentrations (Figure 1F).

To test whether physiologically relevant redox buffer systems can induce the formation of polydisperse HMW assemblies we investigated the effects of different ratios of GSH/GSSG, Cys/CySS and the thioredoxin (Trx) system on the assembly of Bri2 BRICHOS monomers into oligomers. We used concentrations and ratios reported to exist in the intracellular (GSH/GSSG, Trx) or extracellular environment (GSH/GSSG; Cys/CySS, Trx) (Table S1) and inverted ratios thereof to study a spectrum of redox conditions. Samples were analyzed by SDS‐PAGE under reducing and non‐reducing conditions, and by native PAGE (Figure 1G and H, and Figure S3). All redox buffer systems tested promote Bri2 BRICHOS monomers to form disulfide‐dependent HMW assemblies, most obvious when incubated with excess of the reducing equivalent. Furthermore, the Trx system consumes NADPH, which is converted to NADP^+^ in a Bri2 BRICHOS monomer concentration‐dependent manner, but not in the presence of a control protein which lacks Cys, indicating that Trx actively reduces disulfide bonds in Bri2 BRICHOS monomers (Figure S3). Trx promotes the formation of Bri2 BRICHOS disulfide‐dependent assemblies at nanomolar concentrations (Figure 1G and H), which are in the range of extracellular Trx concentrations (Table S1). Incubation of Bri2 BRICHOS monomers and oligomers with the antioxidant/reductant ascorbic acid (vitamin C) had no effects on their assembly state (Figure S3). Our results demonstrate that even mildly reducing conditions induce structural changes in Bri2 BRICHOS monomers that lead to the association of subunits into large, polydisperse and disulfide‐linked oligomers with an increased stability and overall surface hydrophobicity.

### 2.2 Inhibition of non‐fibrillar protein aggregation by reduction‐induced Bri2 BRICHOS oligomers

**FIGURE 2:**
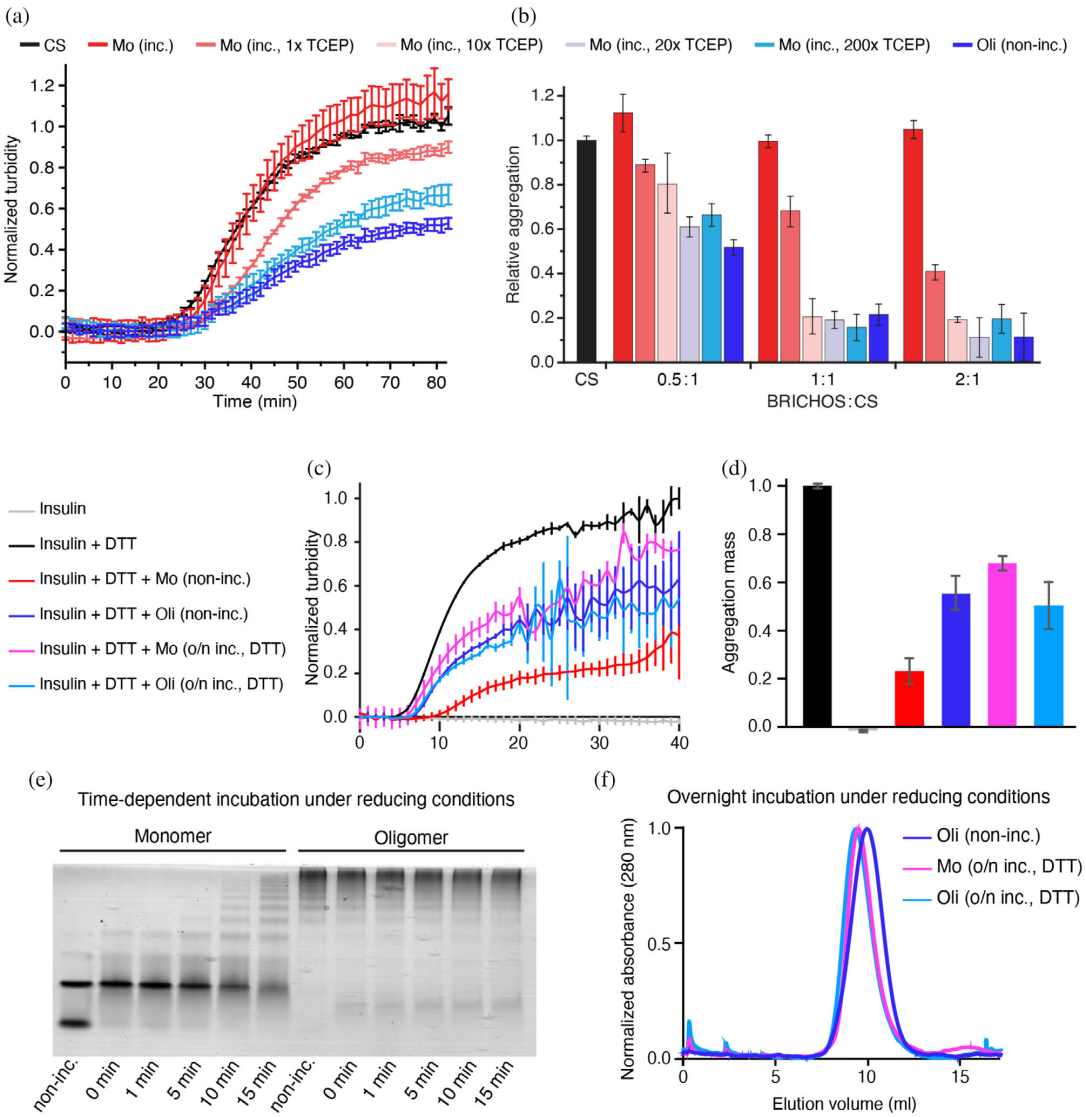
Inhibition of non‐fibrillar protein aggregation by Bri2 BRICHOS assemblies. Bri2 BRICHOS samples are marked as: non‐incubated (non‐inc.) or incubated in the absence or in the presence of reductant (inc.). (a) Aggregation kinetics of 0.6 μM thermo‐denatured CS alone or in the presence of 0.3 μM of different Bri2 BRICHOS samples. (b) CS aggregation at different BRICHOS:CS molar ratios. Values are presented as mean ± *SD* of 3–4 replicates. (c) Aggregation kinetics of 80 μM insulin incubated in the presence of DTT and with or without 5 μM non‐incubated Bri2 BRICHOS monomers or oligomers as well as after overnight incubation with DTT prior to the experiment. (d) Effects of samples in (c) normalized to the aggregation mass which was determined from the areas under the curves. Values are presented as mean ± *SD* of 3 replicates. (e) Native PAGE of Bri2 BRICHOS monomers and oligomers incubated with DTT at different time points. (f) SEC profiles of Bri2 BRICHOS monomers and oligomers after overnight incubation with DTT and non‐incubated oligomers. These samples were used for subsequent analysis in the insulin aggregation assay shown in (c).

To probe the molecular chaperone functions of reduction‐induced Bri2 BRICHOS assemblies, we measured the aggregation behavior of thermo‐denatured citrate synthase (CS) in the presence of different Bri2 BRICHOS species (Figure 2A and B). Oligomers formed from Bri2 BRICHOS monomers during incubation in the presence of TCEP more efficiently prevent CS from aggregation compared to Bri2 BRICHOS monomers incubated without TCEP. In contrast to reducing conditions, strongly oxidizing conditions did not affect the assembly state, overall secondary structures, or the abilities to inhibit CS aggregation of Bri2 BRICHOS monomers or oligomers (Figure S4).

Reduction‐induced Bri2 BRICHOS assemblies are thus efficient molecular chaperones against heat‐induced aggregation of the model substrate CS. Next, we wondered if Bri2 BRICHOS assembly and gain of chaperone function can occur under reducing conditions that lead to misfolding of extracellular proteins. First, we used the well‐established model substrate insulin, which aggregates under reducing conditions, and followed the change in turbidity over time in the absence and presence of Bri2 BRICHOS monomers and oligomers isolated from *E. coli*. Bri2 BRICHOS monomers and oligomers decreased insulin aggregation at sub‐stoichiometric concentrations and in a concentration‐dependent manner (Figure 2C and D and Figure S5). Surprisingly, Bri2 BRICHOS monomers are the most efficient species, which is counterintuitive to our previous findings where only oligomers appeared as active molecular chaperones. We speculated that (1) monomers rapidly convert into HMW assemblies during sample preparation and the lag‐phase of the assay due to the overall reducing assay conditions, and (2) that the higher efficiency starting from monomers compared to oligomers reflect differences in the size of Bri2 BRICHOS oligomers formed and hence affect the stoichiometry of BRICHOS molecules and the substrate. Indeed, short incubation (on a minute timescale) of Bri2 BRICHOS monomers under the same conditions as used in the insulin aggregation assay, rapidly induced polydisperse oligomer formation but with smaller sizes compared to non‐incubated oligomers (Figure 2E). Furthermore, after overnight incubation in the presence of reductant monomers fully converted into oligomers similar in size to *E. coli* purified and non‐incubated oligomers (Figure 2F). Oligomers formed from monomers during overnight incubation were less efficient in preventing insulin aggregation than those formed from monomers during the time course of the aggregation assay but were very similar in activity compared to non‐incubated oligomers (Figure 2C,D). This suggests that monomers can convert rapidly into HMW assemblies during the aggregation assay and that the size of the formed oligomers and hence the stoichiometry of assemblies vs. substrate affect the efficiency.

**FIGURE 3:**
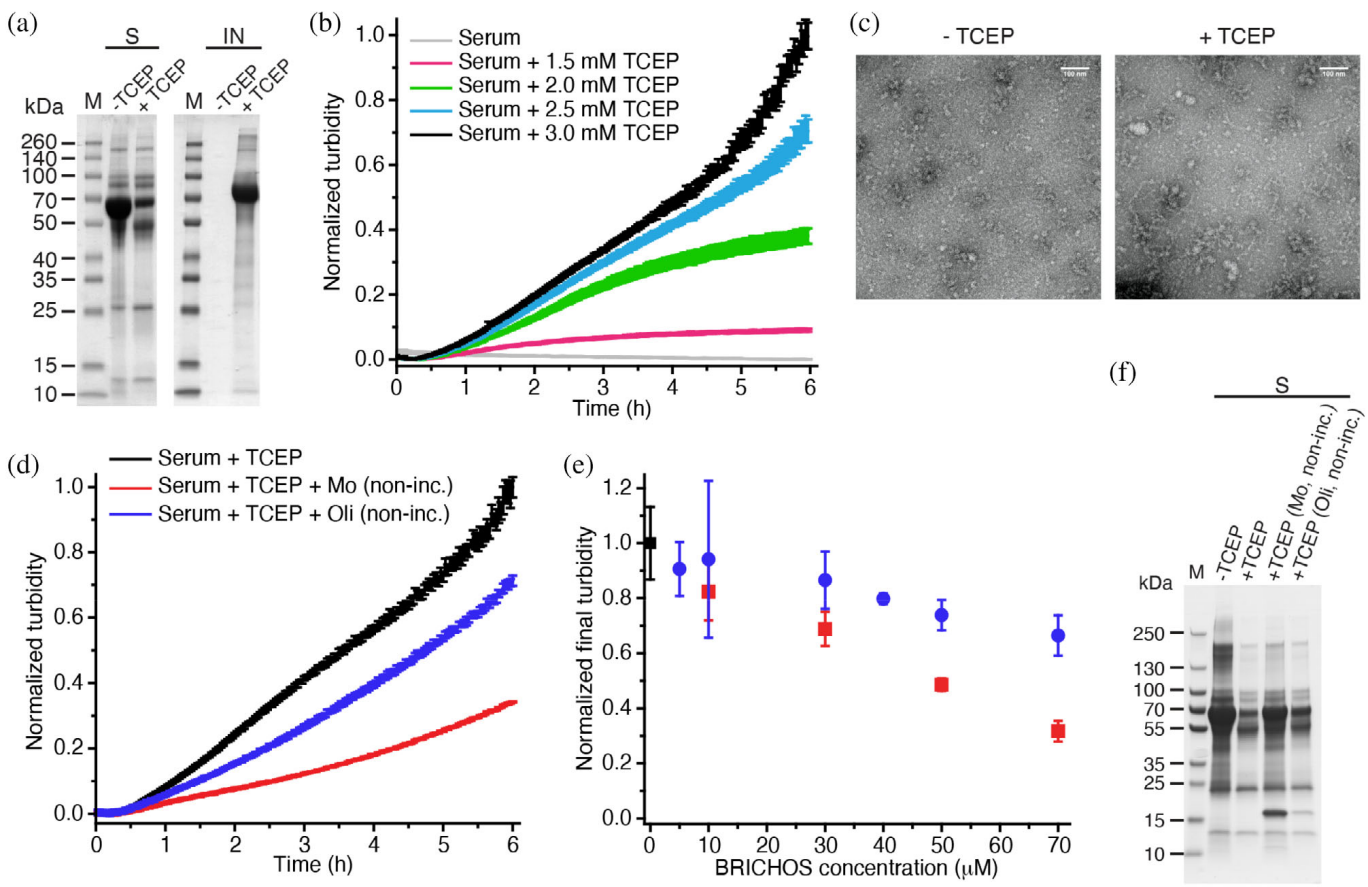
Reduction‐induced serum aggregation and activities of the Bri2 BRICHOS domain. (a) SDS‐PAGE of soluble (S) and insoluble (IN) protein fractions after incubation of rabbit serum in the absence and presence of 3 mM TCEP. (b) Aggregation kinetics of rabbit serum with increasing TCEP concentrations. (c) Transmission electron micrographs of negatively stained serum protein aggregates. (d) Reduction‐induced aggregation of rabbit serum in the absence and in the presence of 70 μM Bri2 BRICHOS. (e) Effects of Bri2 BRICHOS on serum aggregation at varying BRICHOS concentrations. Coloring as in (d). (f) SDS‐PAGE analysis of soluble (S) protein fractions before and after reduction‐induced aggregation of rabbit serum with and without Bri2 BRICHOS. Data in (b), (d) and (e) have been normalized to the endpoint turbidity of serum incubated with TCEP and values in (e) are presented as mean ± *SD* of 3–4 replicates.

Next, we established an additional extracellular protein aggregation assay using serum proteins as model substrate. Serum was incubated at elevated temperatures, in the absence or presence of the reductant TCEP (Figure 3). Serum proteins appear in the insoluble fraction only if reductant is present during heat‐stress and we were able to follow the aggregation kinetics in a TCEP concentration‐dependent manner (Figure 3A,B). As serum protein aggregation might be associated with the formation of amyloid‐like structures, the fluorescent probe Thioflavin T (ThT) was used to measure kinetics of amyloid fibril formation *in vitro*.^17^ ThT‐positive aggregates were observed early during aggregation and the fluorescence signal reached a plateau after ~45 min (Figure S6). Thus, the turbidity due to protein aggregation started to increase significantly after the ThT signal reached a plateau, indicating that the major contribution to serum protein aggregation originates from non‐fibrillar protein aggregates. In line with these findings, aggregates that are observed by negative staining in transmission electron microscopy (TEM) are amorphous (Figure 3C).

Bri2 BRICHOS and α‐crystallin (contains αA‐ and αB‐crystallins) were able to prevent reduction‐induced serum protein aggregation, while the highly soluble non‐chaperone control protein NT*^18^ was completely inefficient (Figure 3D and Figure S6). In accordance with our previous findings, non‐incubated Bri2 BRICHOS monomers which assemble into oligomers during the time course of the assay were more efficient in keeping serum proteins soluble compared to preformed oligomers (Figure 3D – F and Figure S6). We did not observe any change in the sigmoidal growth behavior when measuring serum protein fibril formation kinetics by ThT fluorescence in the presence of any tested protein, only a small change in the final ThT signal intensity (Figure S6). These results show that Bri2 BRICHOS monomers rapidly assemble into oligomers that function as molecular chaperones under reducing conditions.

### 2.3 Mechanism for reduction‐induced chaperone generation

**FIGURE 4:**
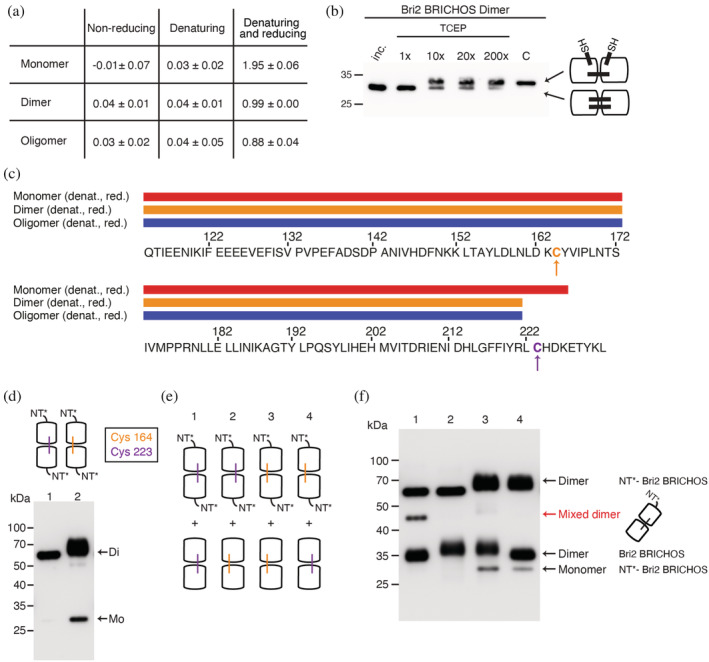
Thiol reactivities and disulfide stabilities of intra‐ and intermolecular disulfide bonds. (a) Quantification of free thiols per molecule in different Bri2 BRICHOS species and in different buffer conditions. Values are presented as mean ± *SD* of 3 replicates. (b) Western blot analysis of the Bri2 BRICHOS dimer band under non‐reducing conditions of incubated Bri2 BRICHOS dimer fractions with increasing molar ratios of TCEP over protein. Lane C corresponds to sample mixed with strongly reducing SDS sample loading buffer and the proposed disulfide states in the dimer are illustrated to the right. (c) MS/MS data analysis after reduction and alkylation of free thiols. Sequence coverage of each species is indicated by colored bars and Cys164 (orange) and Cys223 (purple) are highlighted with arrows. (d) Western blot analysis of dimer fractions of NT*‐Bri2 BRICHOS*cys* mutants (NT*‐Bri2 BRICHOS*cys* 223–223 dimer (1) and NT*‐Bri2 BRICHOS*cys* 164–164 dimer (2)) under non‐reducing conditions. (e) Mixing scheme of different NT*‐Bri2 BRICHOS*cys* and Bri2‐BRICHOS*cys* mutants. (f) Western blot analysis of samples shown in (e). Formation of mixed disulfides *that is,* one molecule NT*‐Bri2 BRICHOS linked with one molecule Bri2 BRICHOS is highlighted in red.

To gain deeper insight into the formation of intermolecular disulfide bonds in the oligomer assembly process, we analyzed the thiol and disulfide content of Bri2 BRICHOS monomers, dimers and oligomers that were isolated from *E. coli* (Figure 4A). No free thiols could be detected for any species under non‐reducing or denaturing (but non‐reducing) conditions. Incubation in denaturing and reducing buffer showed that the intramolecular disulfide bond of the monomer can be completely reduced, resulting in two free thiols per molecule, while for the dimer and oligomer, only approximately one free thiol/molecule is generated. This suggests that the intermolecular disulfide bonds involving two (dimer) or more (HMW assembly) Bri2 BRICHOS subunits have very different redox potentials and/or accessibilities toward reducing agents.

Consequently, we added increasing TCEP concentrations to isolated Bri2 BRICHOS species (*i.e.,* monomers, dimers, and oligomers) and analyzed their migration on SDS‐PAGE under non‐reducing conditions. Two bands appear around the expected size of the 30 kDa dimer at intermediate TCEP concentrations, suggesting that the dimer can exist in a fully oxidized compact conformation with two intermolecular disulfides and a half‐reduced conformation, held together by one disulfide bond (Figure 4B). Interestingly, the monomer and oligomer fractions could be completely reduced by adding a high excess of reductant (Figure S7), while purified dimers resist complete reduction of disulfide bonds under all conditions tested (Figure 4B).

To investigate if different Bri2 BRICHOS species are linked by homo‐ or heterodisulfide bonds, isolated monomers, dimers, and oligomers were cleaved with trypsin and analyzed using mass spectrometry (MS). When digestion was performed under non‐reducing conditions, Cys164‐Cys164 homodisulfide and Cys164‐Cys223 heterodisulfide‐linked peptide fragments were readily detected in all three isolated Bri2 BRICHOS species (Figure S7). In contrast, fragments corresponding to Cys223‐Cys223 homodisulfide‐linked peptids could not be observed. One explanation for the lack of detection of the expected Cys223‐Cys223 peptide fragment (L_222_CHDK_226_) is that it is lost during MS sample preparation and purification, and the concentration thus is below the MS detection limit. However, we were able to detect low‐abundance ions corresponding to a reduced pentapeptide (residues 222–226) which contains the Cys223 in thiol form (Figure S7) indicating that high charge density of this peptide results in low stability of the disulfide‐linked form under the tested conditions.

Under denaturing and reducing conditions (including alkylation of free thiols), we could assign tryptic peptides for both Cys (at positions 164 and 223) only in Bri2 BRICHOS monomer preparations, while we were not able to detect Cys223 containing fragments for dimers and oligomers (Figure 4C). In addition, the only Cys223‐containing fragment in the monomer preparations included a missed cleavage after the neighboring Arg221. The lack of detection of the Cys223 fragment in dimers and oligomers strongly suggest that the C‐terminal intermolecular disulfide bond in Bri2 BRICHOS assemblies is highly resistant to reducing conditions, rendering it inaccessible to tryptic digestion.

To further study the interconnection of dimeric subunits in the oligomer, we selectively substituted Cys residues in Bri2 BRICHOS with Ser and purified mutant Bri2 BRICHOS*cys* dimer fractions with and without the NT* fusion tag as a detection tool. These dimers are by necessity linked by homodisulfide bonds, *that is,* Cys164‐Cys164 or Cys223‐Cys223. Western blotting analysis under non‐reducing conditions revealed that homodisulfide bonds are readily formed and that Cys164 dimers, but not Cys223 dimers, partially dissociate during sample preparation (Figure 4D). This indicates that both Bri2 BRICHOS*cys* constructs are able to form homodisulfides to a large extent and that Cys223 dimers are more readily formed and/or are more stable. To answer the question if homo‐ or heterodisulfides can form between different Bri2 BRICHOS*cys* dimers, we incubated mixtures of the two dimeric Bri2 BRICHOS*cys* proteins with and without the NT* tag, to enable differentiation of the species by their molecular weight on non‐reducing SDS‐PAGE (Figure 4E and F). We detected a band representing the formation of mixed disulfides (*i.e*., one molecule NT*‐Bri2 BRICHOS linked with one molecule Bri2 BRICHOS) for both Cys mutants; however, the band for C164 mixed dimers appears very weak. This shows that both Cys164‐Cys164 and Cys223‐Cys223 are able to mediate disulfide reshuffling when two dimers associate, but Cys223‐Cys223 dimers are more readily reshuffled and/or are more stable. Complete absence of disulfide bonds, in the NT*‐Bri2 BRICHOS double Cys mutant, makes the Bri2 BRICHOS domain highly sensitive to proteolytic digestion during expression (Figure S8). These results highlight the importance of disulfide bond formation for the stability of the Bri2 BRICHOS domain.

## 3 DISCUSSION

Oligomer dynamics of ATP‐independent molecular chaperones are important for substrate recognition. Their activation often involves a shift of the equilibrium toward smaller species, or remodeling of the dimeric substructure in oligomers.^19^, ^20^ Intriguingly, the Bri2 BRICHOS domain gains molecular chaperone functions against non‐fibrillary aggregating substrates through the formation of polydisperse HMW assemblies triggered by reducing conditions, while oxidizing conditions have no effects on structure and function. Importantly, our data demonstrate that Bri2 BRICHOS assembly into chaperones occur simultaneously with reduction‐induced unfolding and aggregation of extracellular proteins.

**FIGURE 5:**
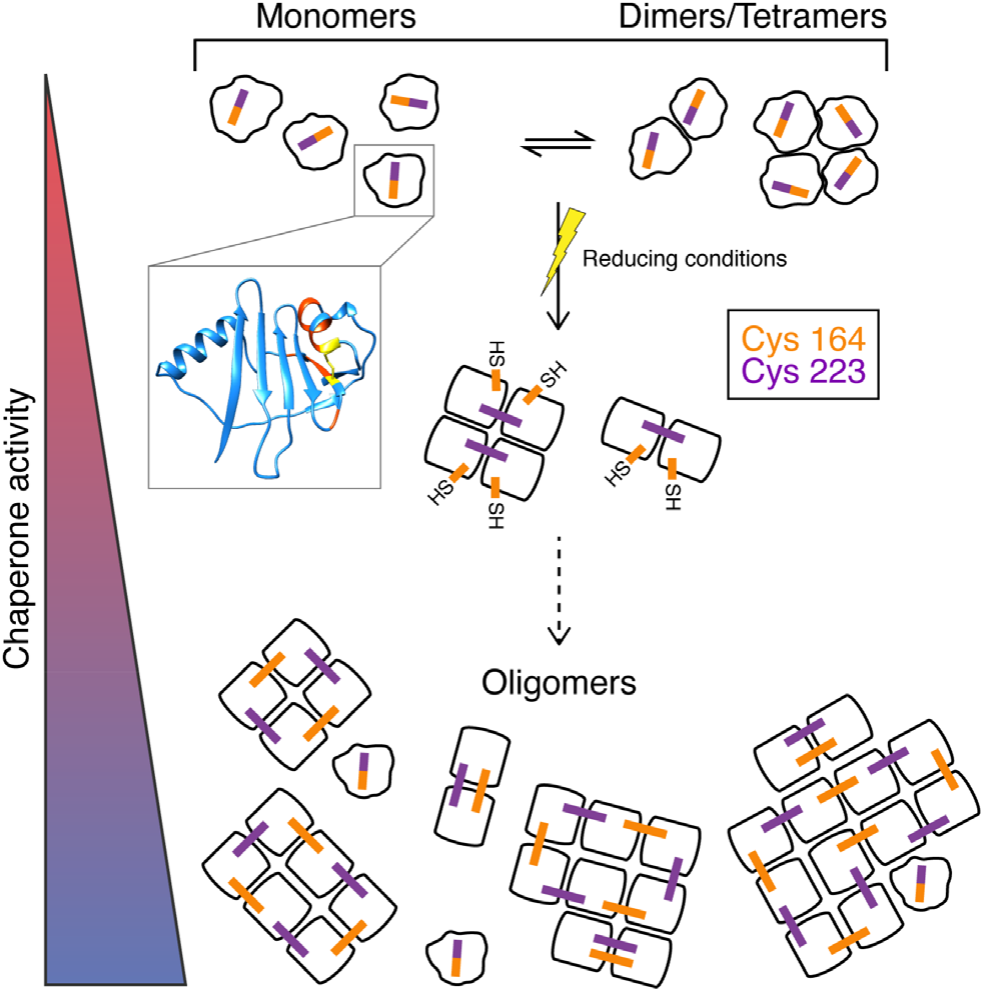
Schematic illustration of the Bri2 BRICHOS assembly process. Bri2 BRICHOS monomers and non‐covalently‐linked dimers and tetramers exist in a dynamic equilibrium. Reduction of the intramolecular disulfide bond in Bri2 BRICHOS monomers (yellow, structural model from the Alphafold database^32^) exposes adjacent hydrophobic stretches (red) and reoxidation of Cys between two monomers leads to the formation of dimers that are stabilized by intermolecular homodisulfide bonds. The Cys223‐Cys223 homodisulfide bond stabilizes the dimer conformation but the Cys223 is also able to swap between two adjacent dimer molecules (see Figure 4). Further growth of the Bri2 BRICHOS HMW assemblies is concluded to be mediated by the Cys164‐Cys164 disulfide. With increasing sizes of the Bri2 BRICHOS HMW assemblies, the chaperone activity against non‐fibrillar substrates increases

Based on disulfide exchange and MS analyses, we show that disulfide bonds are reshuffled during oligomer formation (Figure 5). When the intramolecular disulfide bond in Bri2 BRICHOS monomers is reduced, hydrophobic residues adjacent to both Cys residues likely get exposed. Homodisulfide bonds are established in the dimer and further HMW assembly formation is mediated by reduction and re‐oxidation of homodisulfide bonds. Our observations imply that the accessibility and/or the reactivity of the Cys164‐Cys164 homodisulfide is higher than the more stable Cys223‐Cys223 but we find that both Cys residues cross‐link subunits. Similar to classical sHSPs and ATP‐independent molecular chaperones,^19^, ^20^, ^21^ Bri2 BRICHOS oligomers are highly polydisperse and hence contain monomers that, in the case of Bri2 BRICHOS, feature an intramolecular heterodisulfide bond.

Oxidative stress conditions activate several chaperones, like the cytosolic molecular chaperone Hsp33 from *E. coli* as well as PrxI and PrxII from *yeast*, conceivably to prevent substrates from oxidation‐induced aggregation in an overall reducing environment.^22^, ^23^ In the case of Bri2 BRICHOS, only reducing conditions affect the assembly state and function while strongly oxidizing conditions have no effects. This oxidation‐inert behavior might contribute to the ability of the Bri2 BRICHOS domain to exist and operate in the overall oxidative extracellular environment and enables generation of molecular chaperone functions against non‐fibrillar protein aggregation already at mildly reducing conditions. Interestingly, the mitochondrial peroxiredoxin mTXNPx from *Leishmania infantum* requires reduction of two intermolecular disulfide bonds in the dimer to assemble into an activatable thermo‐sensitive chaperone dodecamer.^24^ Furthermore, calreticulin, an ER‐resident molecular chaperone prevents intracellular (pro)insulin aggregation upon reductive stress insults.^25^ Together, this suggests that molecular chaperone function, as well as their recruitment, is tailored toward the needs and threats of different cellular compartments. To our knowledge, the Bri2 BRICHOS domain is the first example of an extracellular reduction‐activated molecular chaperone.

Although previously considered as redox‐inert, there is increasing evidence that the extracellular environment is dynamically regulated through small molecule redox buffer systems, similar to cellular compartments.^26^ Changes in redox equilibria toward the reductive state might then lead to reductive stress and protein aggregation, increasing the need for chaperoning under these conditions. Indeed, reductive stress has been linked to several pathological conditions including hypoxia, inflammation, and cardiomyopathy with protein aggregation.^27^, ^28^, ^29^ We hypothesize that monomeric Bri2 BRICHOS functions as a homeostatic molecular chaperone against fibrillar (amyloid) aggregation (and might have other, yet unknown, functions) under basal conditions in the oxidizing environment of the extracellular space and can be recruited to prevent amorphous aggregation under reducing conditions. This is supported by previous findings showing that Bri2 BRICHOS monomers are highly efficient inhibitors of fibrillar protein aggregation and associated toxicity but have no chaperone functions against non‐fibrillar protein aggregation.^8^, ^30^ The detailed physiological roles of the Bri2 BRICHOS molecular chaperone under different conditions remain to be established. However, our results highlight the potential of the Bri2 BRICHOS domain as a regulator of reduction‐induced protein aggregation in an overall oxidative environment and pave the way for further mechanistic understanding of extracellular ATP‐independent molecular chaperones.

## 4 MATERIALS AND METHODS

### 4.1 Site‐directed mutagenesis, expression, and purification

The previously described vector pT7HisNT*‐Bri2 BRICHOS^8^ containing the Bri2 BRICHOS sequence (corresponding to residues 113–231 of full‐length human Bri2) was subjected to Cys to Ser mutations (C164S and C223S) using the QuickChange XL site‐directed mutagenesis kit (Agilent Technologies, CA, USA). Expression and purification of the different constructs, including the Bri2 BRICHOS Thr to Trp (T206W) mutant, and their assembly states have been carried out as previously described.^8^, ^14^

### 4.2 Concentration‐dependent assembly formation and Trp fluorescence

Different Bri2 BRICHOS monomer concentrations have been analyzed by SEC using a Superdex 75 PG column. Chromatograms have been normalized to their individual concentration. Individual peaks were fitted with a Gaussian function, the area under the peaks integrated, and the ratio between the amount of Bri2 BRICHOS assemblies (dimers and tetramers) and the total protein amount was calculated and plotted against the initially injected Bri2 BRICHOS concentration. Trp fluorescence experiments were caried out using an Infinite M1000 plate reader (Tecan, Switzerland). 200 μL samples were prepared in a 96 well plate, excited at 280 nm using 5 μm bandwidth, and the fluorescence emission was recorded from 300–400 nm with a 10 μm bandwidth and 1 nm intervals. Data points were fitted with a single exponential function.

### 4.3 Quantification of free thiols

Bri2 BRICHOS species were prepared in: 100 mM Tris, pH 8 (non‐reducing), 6 M Gdn‐HCl in 100 mM Tris, pH 8 (denaturing) or denaturing buffer with 20‐fold molar excess of tris(2‐carboxyethyl)phosphine (TCEP) (reducing). Mixtures were incubated for 16 h at RT and transferred into degassed denaturing buffer by two rounds of buffer exchange using PD‐10 desalting columns (GE Healthcare, UK). Free thiols were quantified by measuring the absorbance change at 412 nm with a spectrophotometer (UV‐1800, Shimadzu) of samples containing 2–4 nmol protein, 300 nmol 5,5‐dithio‐bis‐(2)‐nitrobenzoic acid (DTNB), and calculating the number of free thiols per molecule using the molar absorption coefficient 14,150 M^−1^ cm^−1^ in Tris buffer and 13,700 M^−1^ cm^−1^ in Gdn‐HCl buffer.

### 4.4 Reduction, oxidation, and disulfide bond formation experiments

All proteins and solutions were freshly prepared in 20 mM phosphate buffer, 0.2 mM EDTA, and the pH adjusted to 8. 20 μM protein was incubated overnight at 37°C with different molar ratios of (I) TCEP, (II) GSH:GSSG, (III) Cys:CySS, and (IV) ascorbic acid or 10 mM H_2_O_2_. Reduction of disulfide bonds in Bri2 BRICHOS by human thioredoxin (hTrx; Sigma, USA) was investigated by measuring the consumption of NADPH (Roche, Germany) by rat thioredoxin reductase (TrxR; Sigma, USA). 20 μL samples of each replicate containing varying concentrations of Bri2 BRICHOS monomers, 2 μM hTrx, 0.15 U TrxR and 0.8 mM NADPH were prepared in black half‐area 384‐well polystyrene microplates with a transparent bottom (Corning, USA) and the absorbance at 340 nm was followed using a FLUOStar Galaxy microplate reader (BMG Labtech, Germany) at 37°C.

For mixed disulfide bond formation, dimer fractions of the Bri2 BRICHOS*cys* mutants were purified with or without NT* solubility tag and mixed in a 1:1 M ratio at a final protein concentration of 10 μM in 20 mM phosphate buffer pH 8 containing 0.2 mM EDTA, 0.02% NaN_3_, 2.5x thrombin inhibitor and incubated at 37°C overnight.

Short‐ and long‐term incubation experiments were performed under the same conditions as in the insulin aggregation assay. For SEC analysis, a Superdex 200 increase 10/300 GL column was used. For analysis of short‐term effects, samples have been rapidly mixed with native PAGE buffer and directly snap frozen in liquid nitrogen before separation by native PAGE. For long‐term incubation, samples have been incubated overnight at 37°C.

### 4.5 Western blot

Samples separated by PAGE were transferred to nitrocellulose membranes (GE Healthcare Life sciences, Amersham, UK), blocked with 5% milk in PBS and incubated with polyclonal goat anti‐Bri2 (raised against recombinant human Bri2 BRICHCHOS 113–231) (1,250 diluted). Polyclonal rabbit anti‐goat IgG/HRP (Thermo Fisher Scientific, USA; 61–1,620) antibody has been used as secondary antibody and the protein bands were detected with the ECL™ Western Blotting detection kit (GE Healthcare, UK; RPN2209) using a CCD camera (Fujifilm LAS‐3000; Japan).

### 4.6 Circular dichroism (CD) spectroscopy

CD spectra were recorded in 1 nm increments, with a 1 nm bandwidth between 190 nm, and 260 nm on a Chirascan (Applied Photophysics, U.K.) using quartz glass cuvettes with a 1 mm path length. The measurements were performed at 25°C in 20 mM phosphate buffer pH 8 containing 10 μM protein. Spectra represent the average of five consecutive scans and the corresponding buffer blanks were subtracted.

### 4.7 Bis‐ANS fluorescence

Samples containing 1 μM protein and 2 μM 4,4’‐Dianilino‐1,1′‐binaphthyl‐5,5′‐disulfonic acid (bis‐ANS) were incubated for 10 min at 25°C. Fluorescence emission spectra were measured with a Fluorolog‐3 (Horiba, Japan) fluorescence spectrometer between 420 and 600 nm at an excitation wavelength of 395 nm. Data thereof represent the average of three individual replicates.

### 4.8 Aggregation assays of citrate synthase (CS), whole rabbit serum, and insulin

The buffer of commercially available α‐crystallin from bovine eye lens (C4163, Sigma, USA) was exchanged to PBS pH 7.4. CS experiments were carried out as described in.^8^ For aggregation experiments with whole rabbit serum (Abcam, ab166640, UK), 35 μL of each sample was prepared in black half‐area 384‐well polystyrene microplates with a transparent bottom (Corning, USA). Each replicate contained a six‐fold dilution of rabbit serum (final concentration: 8.9 ± 0.6 mg/mL) in PBS pH 7.4, 3 mM TCEP (if not stated differently), and different Bri2 BRICHOS, NT* or α‐crystallin concentrations. Serum concentrations were determined by the BCA method. Assuming an average molecular weight range of 60–80 kDa for serum proteins, the serum protein concentration in the assay ranged from 150–110 μM. Aggregation kinetics were measured during incubation at 45°C and the apparent change in the absorbance at 360 nm recorded with a FLUOStar Galaxy microplate reader (BMG Labtech, Germany). To measure ThT fluorescence, samples were additionally supplemented with 10 μM ThT and measurements performed with a 440 nm excitation filter and a 480 nm emission filter. For insulin aggregation assays, a 1.5 mM stock of insulin was prepared in 1x PBS and the pH adjusted to 7.4. Aggregation was initiated by addition of 10 mM DTT to 80 μM insulin and incubation at 37°C. Sample preparation and instrumentation were the same as previously described. For preincubation, 20 μM BRICHOS were incubated in the presence of 10 mM DTT overnight at room temperature and excess of DTT removed prior to the aggregation assay by SEC.

### 4.9 Transmission electron microscopy

4 μL of rabbit serum incubated with or without 3 mM TCEP for 6 h at 45°C was adsorbed onto carbon‐coated copper grids (400 mesh, Analytical Standards). Negative staining was done with uranyl acetate [2% (w/v)] for 45 sec. The samples were imaged using a JEOL JEM2100F field emission gun transmission electron microscope (JEOL, Japan) operating at 200 kV.

### 4.10 Mass spectrometry experiments (LC–MS/MS)

60 μg protein were lyophilized and dissolved in 8 M Urea in 20 mM Tris buffer pH 8 with or without 10 mM DTT. Samples were heated at 56°C for 20 min and 20 mM Idodacetamide added for 30 min incubation in the dark at RT. Samples were diluted with 20 mM Tris buffer pH 8 to a final concentration of 0.5 M Urea and 1:20 (w/w) and MS grade trypsin protease (Thermo Scientific, USA) was added for incubation overnight at 37°C. Samples were purified using a solid phase extraction (SPE‐SCX strata‐X‐C, Phenomenex), dried, and resuspended in 3% acetonitrile with 0.1% formic acid. Online LC–MS was performed using a Dionex UltiMate™ 3,000 RSLCnano System coupled to a Fusion mass spectrometer (Thermo Scientific). Samples were dissolved in 20 μL solvent A and 10 μL were injected. Samples were trapped on a C18 guard‐desalting column (Acclaim PepMap 100, 75 μm x 2 cm, nanoViper, C18, 5 μm, 100 Å), and separated on a 50 cm long C18 column (Easy spray PepMap RSLC, C18, 2 μm, 100 Å, 75 μm x 50 cm). The nano capillary solvent A was 99.9% water and 0.1% formic acid; solvent B was 5% water, 95% acetonitrile, and 0.1% formic acid. At a constant flow of 0.25 μL min^−1^, the gradient went from 6–8% B up to 40% B in 20 min, followed by a steep increase to 100% B in 5 min. FTMS master scans with 60,000 resolution (and mass range 300–1,500 m/z) were followed by data‐dependent MS/MS (30,000 resolution) on the top 5 ions using higher energy collision dissociation (HCD) and collision induced dissociation (CID) at 35% normalized collision energy. Precursors were isolated with a 2 m/z window. Automatic gain control (AGC) targets were 1x10^6^ for MS1 and 1x10^5^ for MS2. Maximum injection times were 100 ms for MS1 and 100 ms for MS2. The entire duty cycle lasted ~2.5 s. Dynamic exclusion was used with 30 s duration. Precursors with unassigned charge state or charge state 1 were excluded. An underfill ratio of 1% was used.

### 4.11 Mass spectrometry data analysis

Samples prepared under reducing conditions were analyzed using the Thermo Scientific Proteome Discoverer 1.4 software (Thermo). MS raw files were searched using SequestHT‐Target Decoy PSM Validator against human Swissprot database (released March 2019) and filtered to a 1% FDR cut off. A precursor ion mass tolerance of 10 ppm, and product ion mass tolerances of 0.02 Da for HCD‐FTMS and 0.8 Da for CID‐ITMS was used. The algorithm considered tryptic peptides with maximum two missed cleavages; carbamidomethylation (Cys) as fixed and oxidation (Met), as variable modification. LC–MS data from samples prepared under non‐reducing conditions were manually searched for precursor masses of disulfide‐linked peptide fragments using the Thermo Xcalibur 3.0.63 software. Recorded MS^2^ peak patterns were matched to theoretically fragmented precursor peptides for HCD and CID from the MS^2^ peak intensity prediction (MS2PIP) web server.^31^

## AUTHOR CONTRIBUTIONS

**Axel Leppert:** Conceptualization (supporting); data curation (lead); formal analysis (lead); funding acquisition (supporting); investigation (equal); methodology (equal); visualization (lead); writing – original draft (lead); writing – review and editing (equal). **Gefei Chen:** Data curation (supporting); formal analysis (supporting); investigation (supporting); methodology (supporting); supervision (equal); writing – review and editing (equal). **Danai Lianoudaki:** Data curation (supporting); methodology (equal); writing – review and editing (supporting). **Chloe Williams:** Data curation (supporting); formal analysis (supporting); methodology (supporting); writing – review and editing (supporting). **Xueying Zhong:** Data curation (supporting); writing – review and editing (supporting). **Jonathan D. Gilthorpe:** Formal analysis (supporting); investigation (supporting); methodology (supporting); writing – review and editing (supporting). **Michael Landreh:** Formal analysis (supporting); investigation (supporting); methodology (supporting); supervision (equal); visualization (supporting); writing – original draft (supporting); writing – review and editing (supporting). **Jan Johansson:** Conceptualization (lead); funding acquisition (lead); methodology (supporting); project administration (lead); resources (lead); supervision (lead); visualization (supporting); writing – original draft (supporting); writing – review and editing (supporting).

## CONFLICT OF INTEREST

The authors declare no competing interests.

## Supporting information


**Figure S1** Concentration‐dependent non‐covalent assembly of Bri2 BRICHOS monomers
**Figure S2** Secondary structure changes upon thermal incubation
**Figure S3** Extended data (reducing SDS‐PAGE) related to Figure 1G and H
**Figure S4** Effects of strongly oxidizing conditions on Bri2 BRICHOS assembly and function
**Figure S5** Extended data (reduction‐induced insulin aggregation) related to Figure 2C
**Figure S6** Extended data related to Figure 3
**Figure S7** Redox state of Bri2 BRICHOS dimers and identification of homo‐ and heterodisulfide‐linked peptide fragments by MS
**Figure S8** Non‐reducing SDS‐PAGE of NT*‐Bri2 BRICHOS double Cys mutant monomers
**Table S1** Reported concentrations of redox buffer systems in the intra‐ and extracellular space

## Data Availability

The data that support the findings of this study are available from the corresponding author upon reasonable request.
