# Supplementary material for: ATP‐independent molecular chaperone activity generated under reducing conditions
Source: Protein Sci. 2022 Jul 13;31(8):e4378. doi: 10.1002/pro.4378 (PMC9278091; doi:10.1002/pro.4378)
Supplement: Supplementary file 1 — Figure S1 Concentration‐dependent non‐covalent assembly of Bri2 BRICHOS monomers Figure S2 Secondary structure changes upon thermal incubation Figure S3 Extended data (reducing SDS‐PAGE) related to Figure 1G and H Figure S4 Effects of strongly oxidizing conditions on Bri2 BRICHOS assembly and function Figure S5 Extended data (reduction‐induced insulin aggregation) related to Figure 2C Figure S6 Extended data related to Figure 3 Figure S7 Redox state of Bri2 BRICHOS dimers and identification of homo‐ and heterodisulfide‐linked peptide fragments by MS Figure S8 Non‐reducing SDS‐PAGE of NT*‐Bri2 BRICHOS double Cys mutant monomers Table S1 Reported concentrations of redox buffer systems in the intra‐ and extracellular space [file PRO-31-e4378-s001.docx]

**Supplementary material**

**ATP-independent molecular chaperone activity generated under reducing conditions**

Axel Leppert^1,2*^, Gefei Chen^1^, Danai Lianoudaki^2^, Chloe Williams^3^, Xueying Zhong^4^, Jonathan D. Gilthorpe^3^, Michael Landreh^2^ and Jan Johansson^1^

^1^Department of Biosciences and Nutrition, Karolinska Institutet, 141 83 Huddinge, Sweden

^2^Department of Microbiology, Tumour and Cell Biology, Karolinska Institutet, 171 65 Solna, Sweden

^3^Department of Integrative Medical Biology, Umeå University, 901 87 Umeå, Sweden

^4^Division of Structural Biotechnology, Department of Biomedical Engineering and Health Systems, School of Engineering Sciences in Chemistry, Biotechnology and Health (CBH), KTH Royal Institute of Technology, 141 83 Huddinge, Sweden

*Corresponding author

**
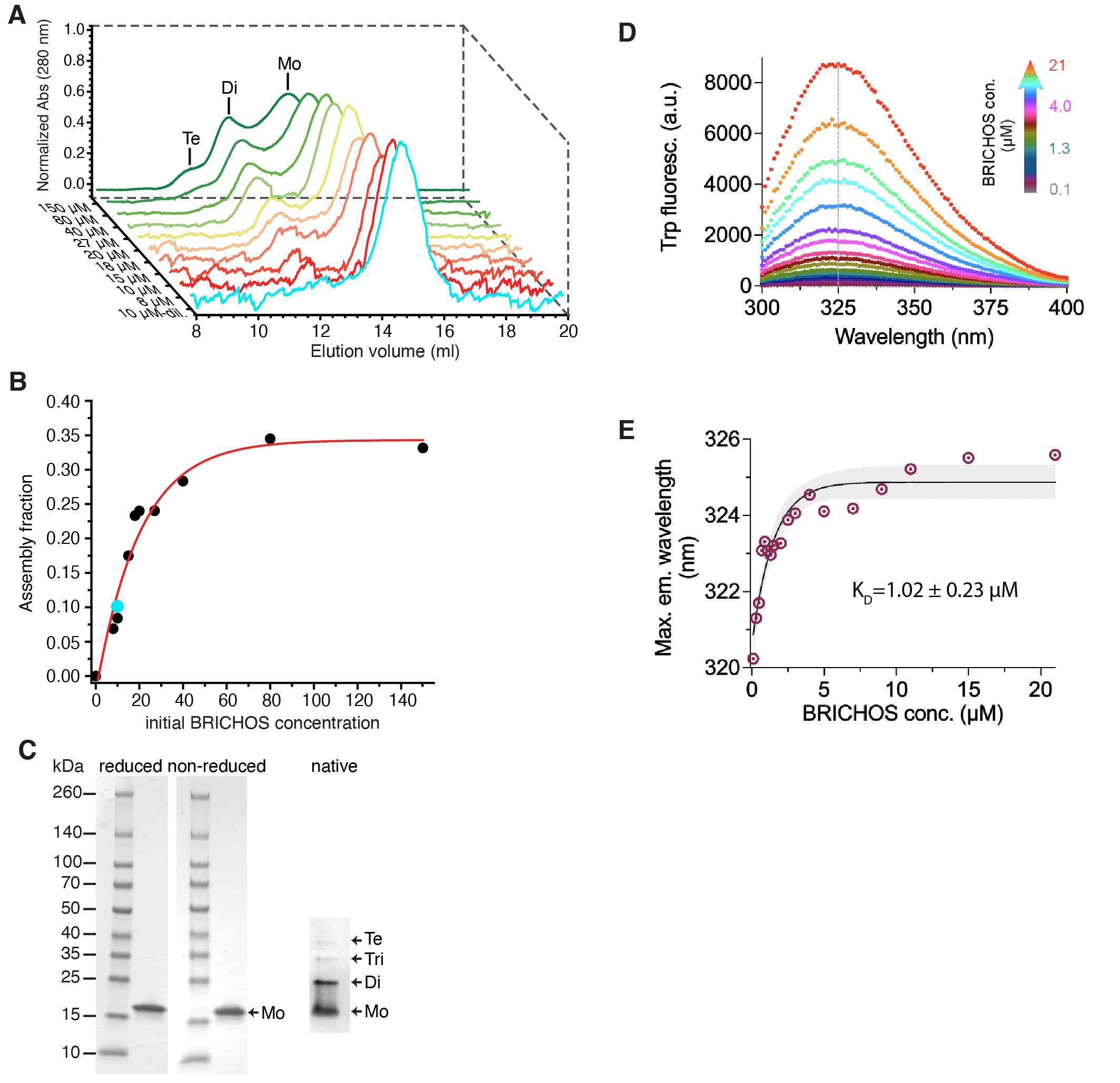
**

**Figure S1. Concentration dependent assembly of Bri2 BRICHOS monomers. (A)** SEC elution profiles of different concentrations of Bri2 BRICHOS monomers isolated from *E. coli* (from dark red to dark green) and after diluting the highest concentrated sample (150 µM) to 10 µM (cyan). The assembly state is indicated at the peak maxima (Mo: monomer; Di: dimer; Te: tetramer). **(B)** Fraction of Bri2 BRICHOS assemblies (dimers and tetramers) plotted against the initially injected concentration. Data points (black) were monoexponentially fitted (red), and the data point of the diluted sample is shown in cyan. For better fitting the data point at 0 was included. **(C)** SDS-PAGE analysis under reducing and non-reducing conditions, and native PAGE of the highest concentrated sample (150 µM)**. (D)** Tryptophan fluorescence emission spectra of Bri2 BRICHOS T206W monomers measured at different concentrations. **(E)** Maximum emission wavelength from (D) plotted against BRICHOS concentrations were fitted with a monoexponential equation, an apparent K_D_ of 1.02 ± 0.23 µM was calculated and the shadow represents the 95% confidence band.


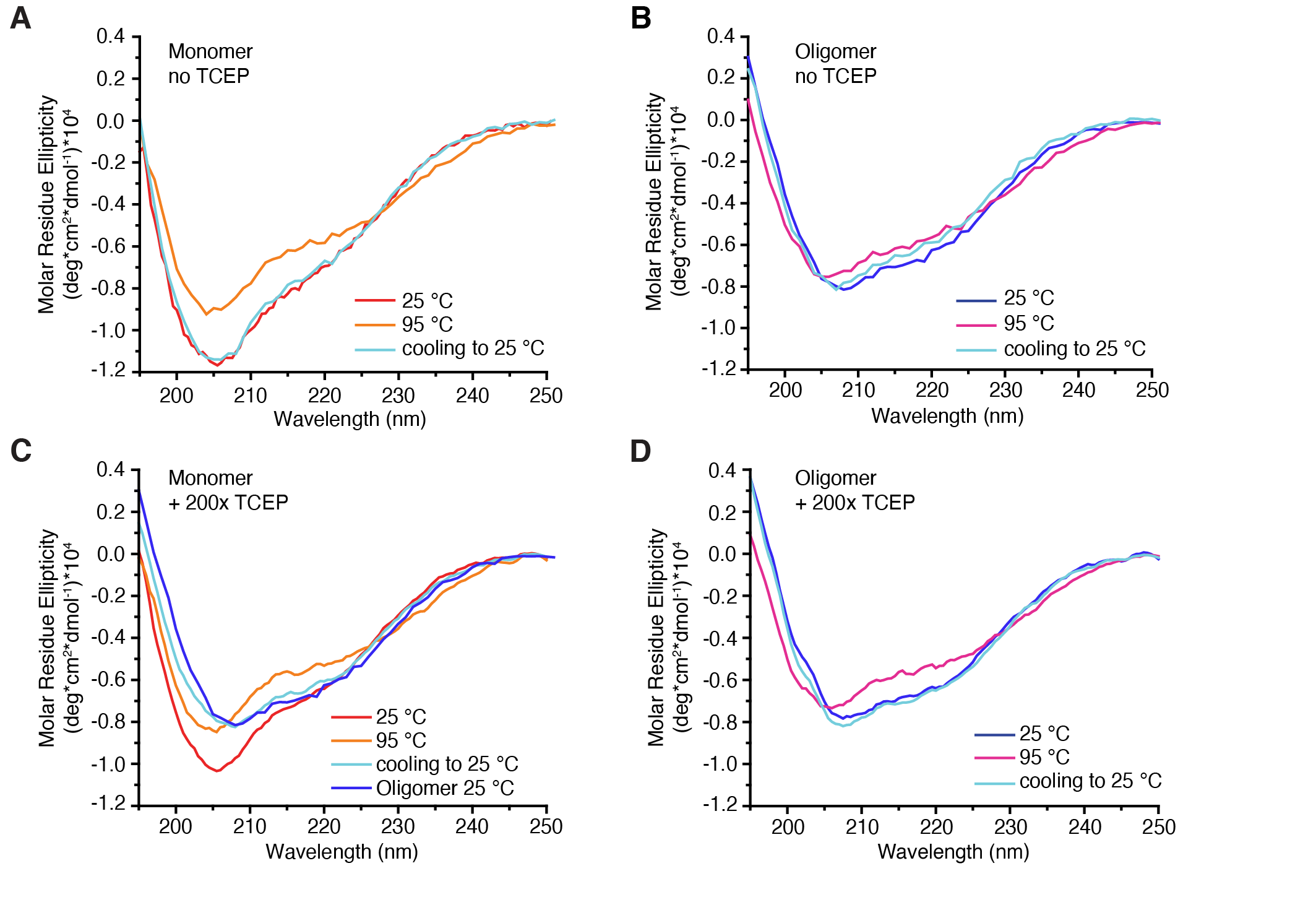


**Figure S2. Secondary structure changes upon thermal incubation measured by far-UV CD spectroscopy**. Bri2 BRICHOS monomers **(A)** and oligomers **(B)** that were isolated from *E. coli* measured at 25°C (red), 95°C (orange), and after cooling to 25°C (light blue) in the absence of TCEP. **(C)** and **(D)** show the same samples as in (A) and (B), respectively but in the presence of a 200-fold TCEP. In (C) *E. coli* isolated Bri2 BRICHOS oligomers that were measured at 25°C are shown for better comparison.

**
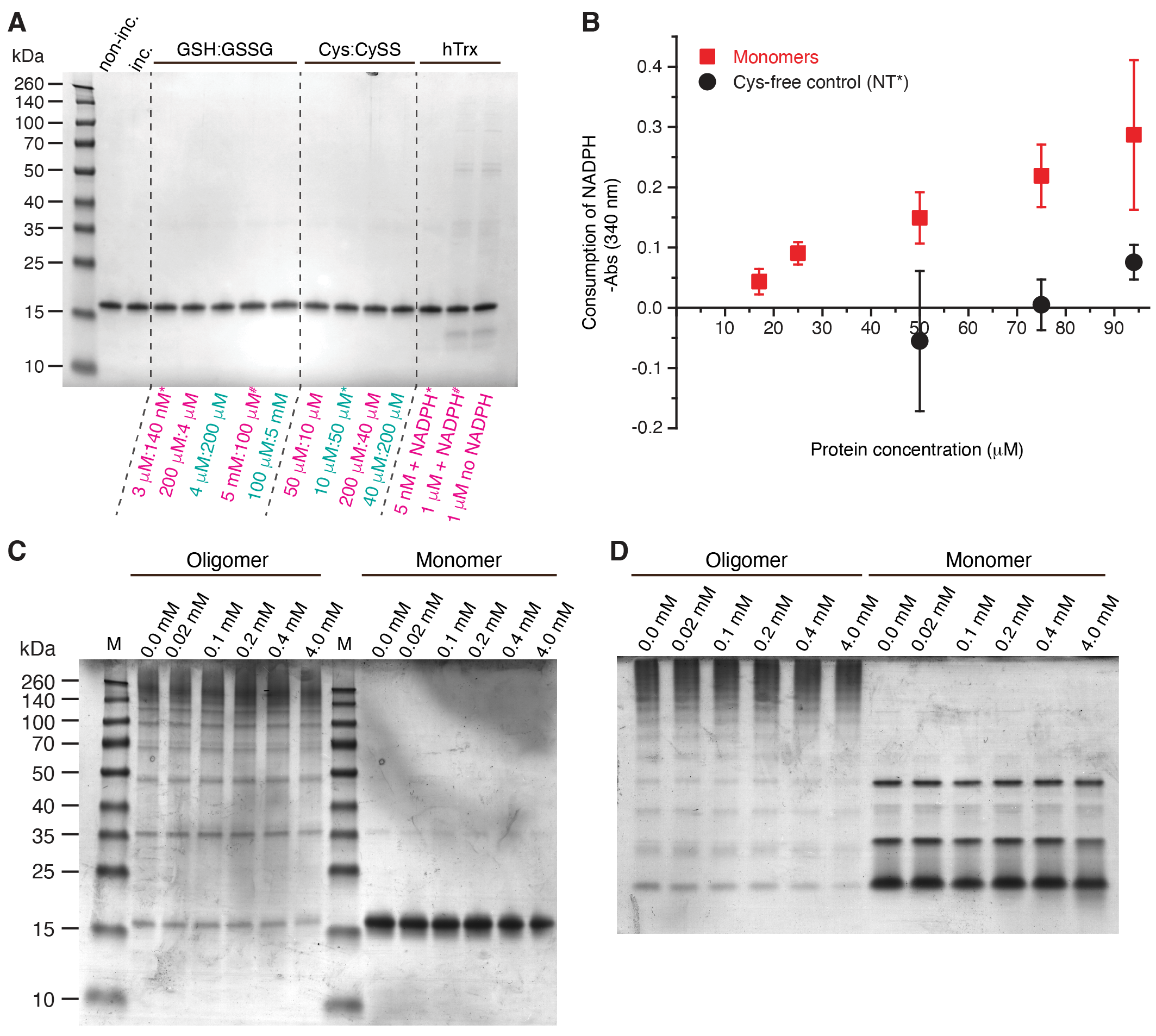
**

**Figure S3. Formation of HMW assemblies mediated by different redox buffer systems and vitamin C.** Bri2 BRICHOS samples are marked as: non-incubated (non-inc.) and incubated in the absence or in the presence of reductant (inc.). **(A)** Reducing SDS-PAGE analysis of Bri2 BRICHOS monomers before and after incubation in the absence (A) and presence of various redox buffer systems as indicated above the lines. The concentrations and molar ratios of the reduced and oxidized variant are indicated below each lane. Excess of the reducing equivalent is highlighted in magenta and excess of the oxidizing equivalent in green. Physiological redox couple concentrations are indicated with # (intracellular) or * (extracellular). **(B)** Trx dependent consumption of NADPH by thioredoxin reductase in the presence of different concentrations of Bri2 BRICHOS monomers (red) or NT* as a Cys-free control protein (black). Data is presented as mean values ± s.d. of 3 independent measurements. SDS-PAGE analysis under non-reducing conditions **(C)** and native PAGE **(D)** of Bri2 BRICHOS monomers or Bri2 BRICHOS oligomers isolated from *E. coli* after incubation in the presence of different concentrations of ascorbic acid (vitamin C) as indicated above each lane.

**
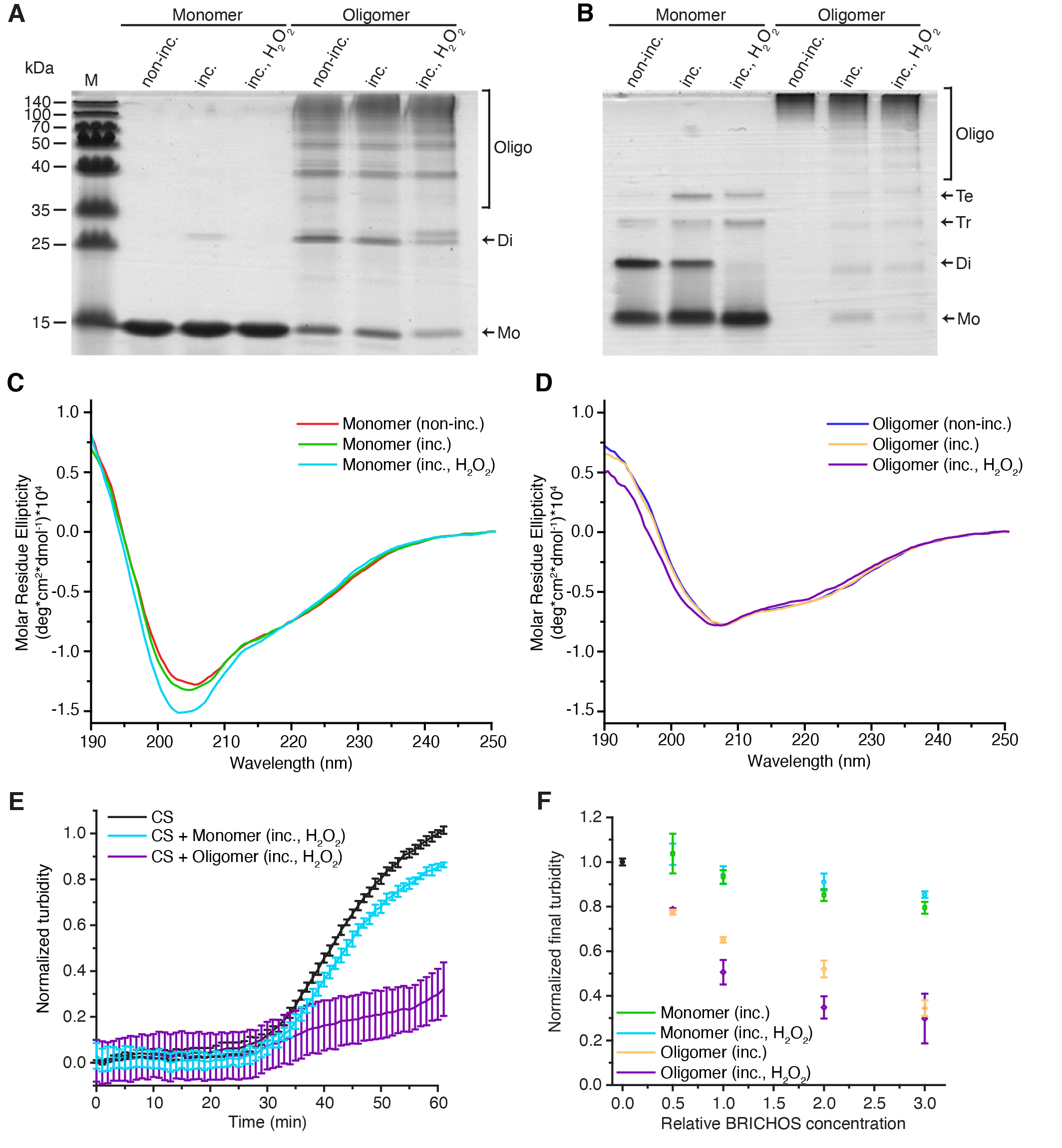
**

**Figure S4. Effects of strongly oxidizing conditions on Bri2 BRICHOS assembly and efficiency to prevent non-fibrillar protein aggregation.** SDS-PAGE analysis under non-reducing conditions **(A)** and native PAGE **(B)** of non-incubated Bri2 BRICHOS monomers or Bri2 BRICHOS oligomers, or after incubation in the absence and presence of 10 mM H_2_O_2_. The assembly states are indicated to the right of the gel (Mo: monomer; Di: dimer; Tr: trimer; Te: tetramer). **(C)** Far-UV CD spectra of non-incubated Bri2 BRICHOS monomers (red) and after incubation in the absence (green) or in the presence of H_2_O_2_ (light blue). **(D)** Far-UV CD spectra of non-incubated Bri2 BRICHOS oligomers (blue) and after incubation in the absence (light orange) or in the presence of H_2_O_2_ (purple). **(E)** Aggregation kinetics of 0.6 µM CS incubated alone (black) and in the presence of 1.8 µM Bri2 BRICHOS monomers (light blue) or oligomers (purple) after incubation with H_2_O_2_. **(F)** Effects of varying Bri2 BRICHOS concentrations on the aggregation of CS. Bri2 BRICHOS monomers after incubation without (green) and with (blue) H_2_O_2_ and Bri2 BRICHOS oligomers after incubation without (light orange) and with (purple) H_2_O_2_. Calculations are based on monomeric subunits. Data has been normalized to the endpoint turbidity of CS alone and values are presented as mean ± s.d. of 4 replicates.


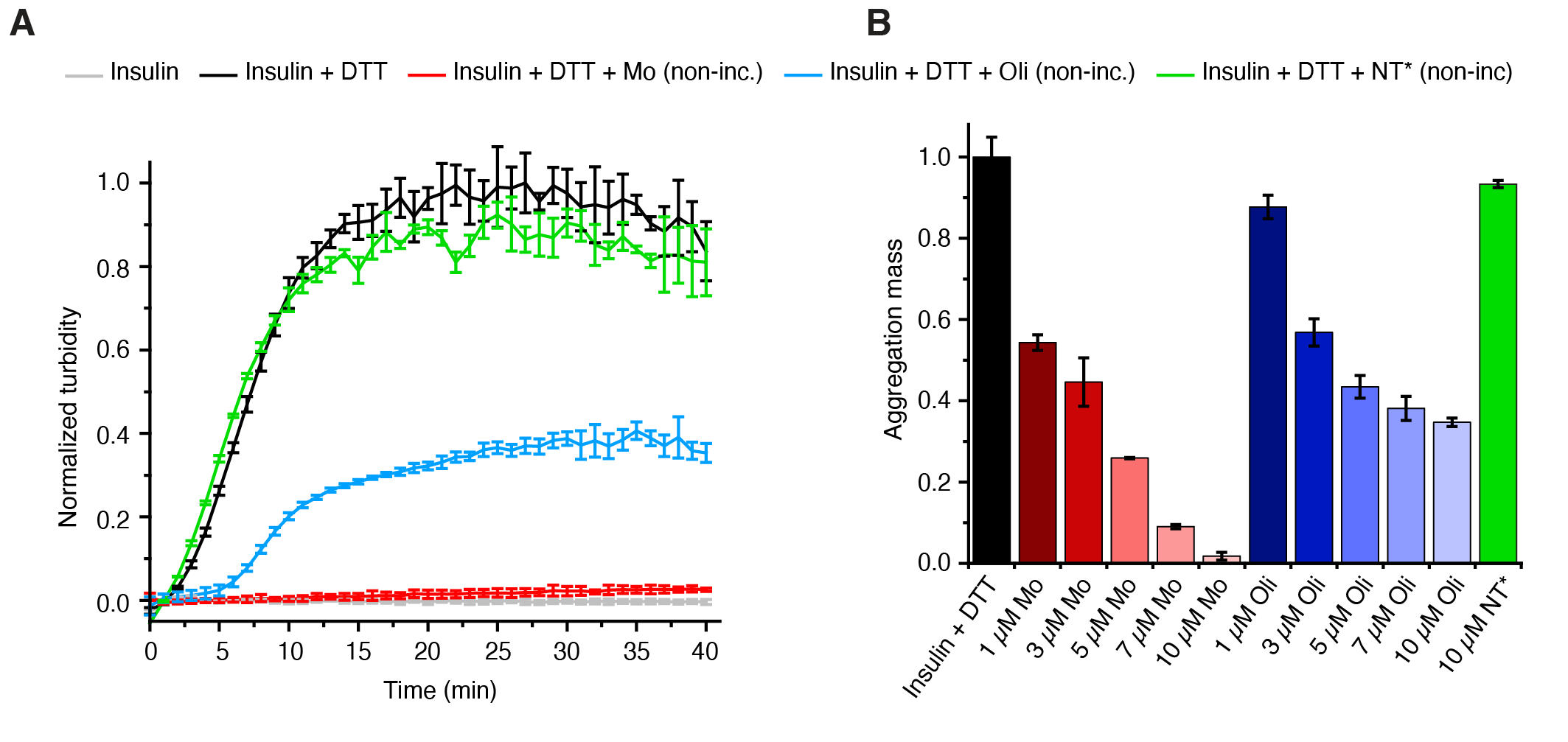


**Figure S5. Activities of Bri2 BRICHOS assemblies against non-fibrillar reduction-induced aggregation of insulin. (A)** Aggregation kinetics of 80 µM insulin incubated in the presence of 10 mM DTT and from *E. coli* isolated 10 µM of Bri2 BRICHOS monomers and oligomers, and the non-chaperone protein NT*. **(B)** Effects of different protein concentrations as indicated on the x-axis on the aggregation of insulin. Data have been normalized to the aggregation mass which was determined from the areas under the curves. Values are presented as mean ± s.d. of 3 replicates.

**
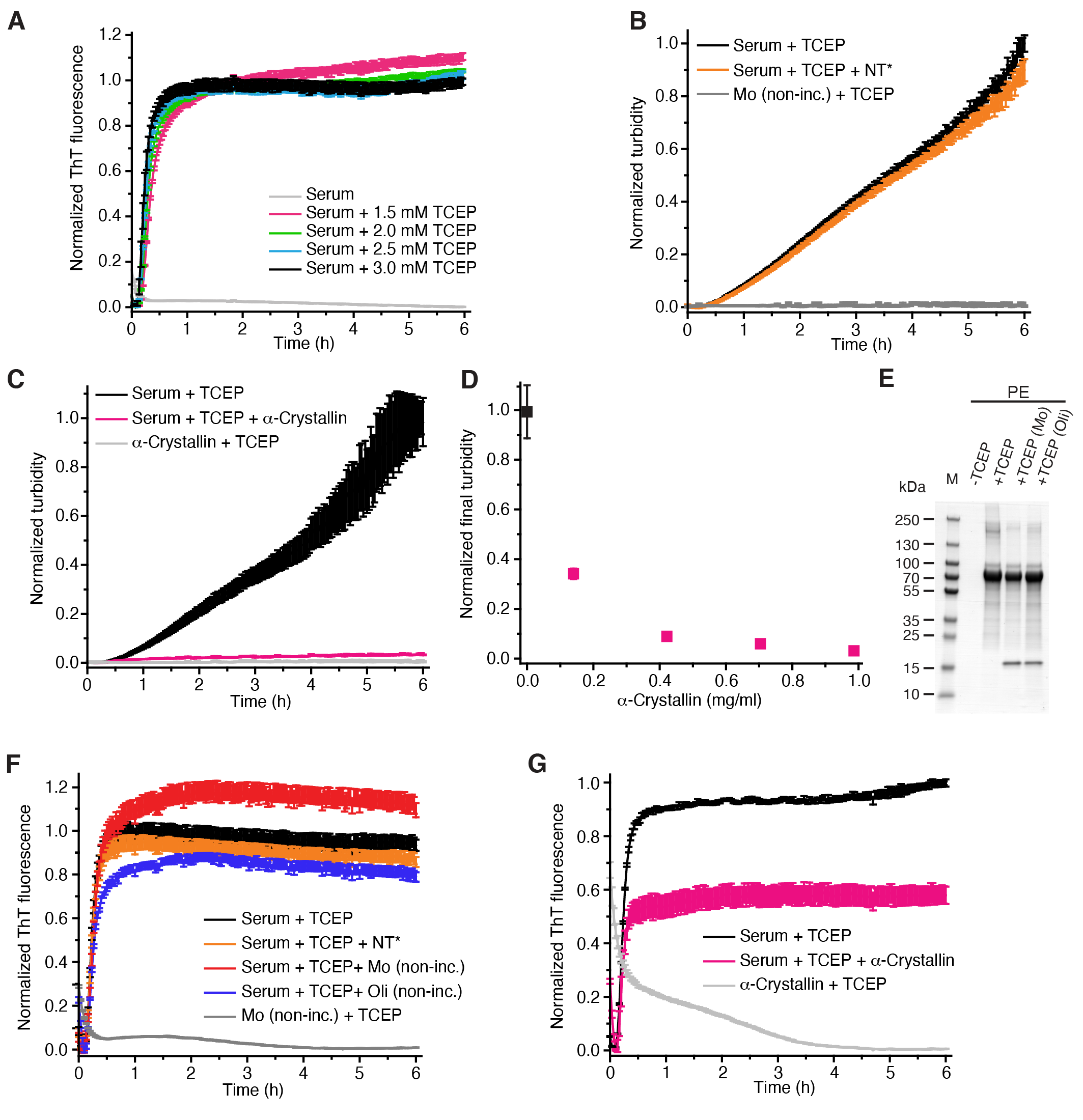
**

**Figure S6. Reduction-induced serum aggregation and effects of Bri2 BRICHOS, α-Crystallin and NT*. (A)** Aggregation kinetics of rabbit serum measured in the presence of ThT without (light grey) and with increasing concentrations of TCEP. Data has been normalized to the maximum ThT fluorescence of serum incubated with 3 mM TCEP. **(B)** 3 mM TCEP-induced aggregation kinetics of rabbit serum during incubation in the absence (black) and in the presence of 100 µM of the non-chaperone protein NT* (orange), as well as 70 µM Bri2 BRICHOS monomers isolated from *E. coli* under the same assay conditions (grey). **(C)** Serum aggregation as in (B) in the presence of 1 mg/ml α-Crystallin (pink) as well as 1 mg/ml α-Crystallin in absence of serum (grey). **(D)** Effects of α-Crystallin on serum aggregation at varying protein amounts. **(E)** SDS-PAGE analysis of insoluble (PE) protein fractions before and after reduction-induced aggregation of rabbit serum with and without Bri2 BRICHOS. **(F)** 3 mM TCEP-induced aggregation of rabbit serum. ThT aggregation traces in the absence (black) and in the presence of 100 µM NT* (orange), 70 µM Bri2 BRICHOS monomers (red), 70 µM Bri2 BRICHOS oligomers (blue) as well as 70 µM Bri2 BRICHOS monomers incubated in the absence of serum (grey). **(G)** ThT aggregation traces as in (E) in the absence (black) and in the presence of 1 mg/ml α-Crystallin (purple) as well as 1 mg/ml α-Crystallin incubated alone (grey). Data has been normalized to the maximum ThT fluorescence of serum incubated with 3 mM TCEP. All data is presented as mean values ± s.d. of 3-4 replicates.

**
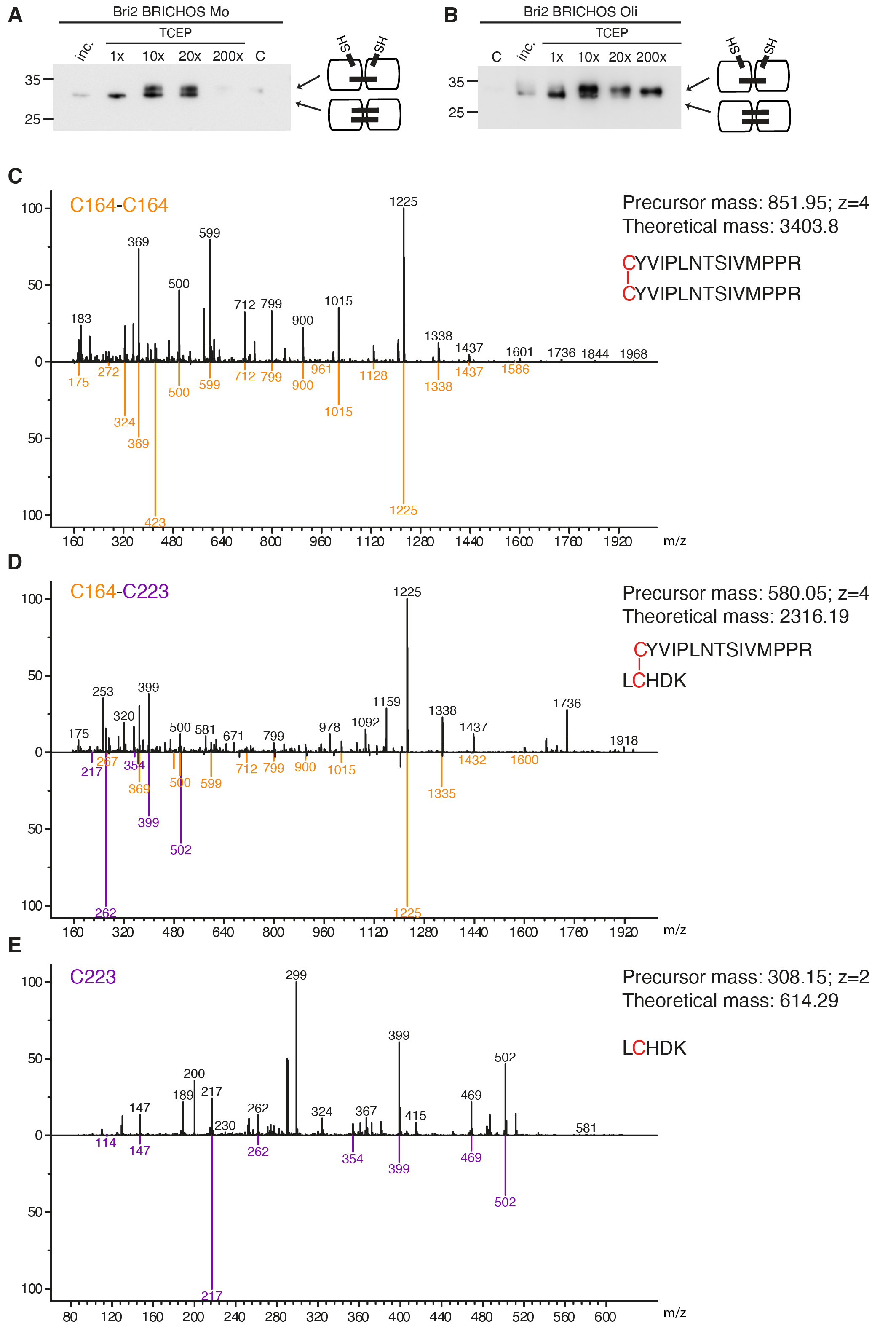
**

**Figure S7. Redox state of Bri2 BRICHOS dimers and identification of homo- and heterodisulfide linked peptide fragments by MS.** Western blot analysis of the Bri2 BRICHOS dimer band under non-reducing conditions of isolated Bri2 BRICHOS monomer **(A)** or Bri2 BRICHOS oligomer **(B)** species after incubation in the presence of different molar ratios of TCEP over protein as indicated above each lane. Lane C corresponds to samples mixed with strongly reducing SDS sample loading buffer SDS buffer. The illustration on right side of the gels indicates the proposed redox state of the two intermolecular disulfide bonds in Bri2 BRICHOS dimers. **(C-E)** Recorded MS^2^ spectra (above center line) and predicted MS^2^ peak intensities (below center line) of peptide fragments linked by a Cys164-Cys164 homodisulfide bond **(C)**, Cys164-Cys223 heterodisulfide bond **(D)** and the peptide fragment containing the reduced Cys223 thiol **(E)**. For each precursor peptide fragment the respective theoretical mass, the detected precursor mass with the corresponding charge state (z), and the sequence of the peptide fragment are shown to the right.


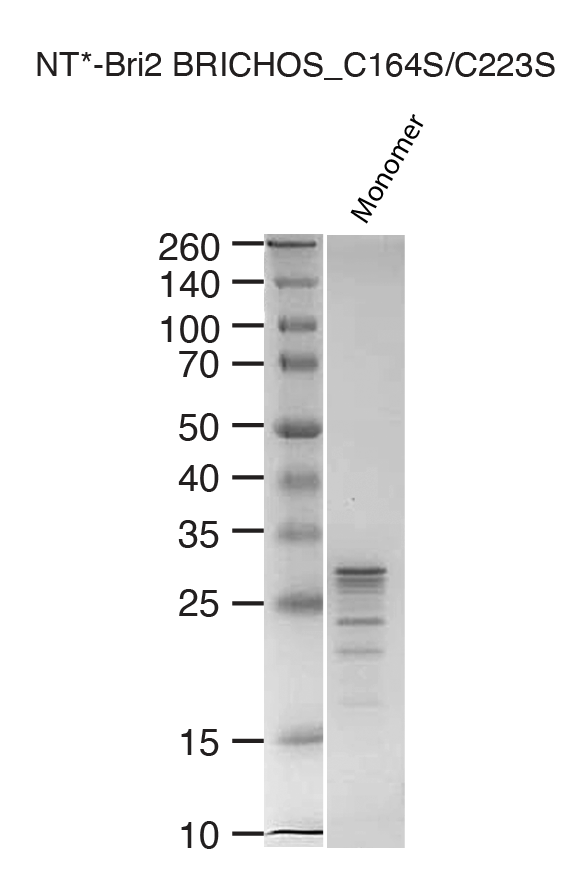


**Figure S8. SDS-PAGE analysis of the Cys-free NT*-Bri2 BRICHOS mutant (C164S/C223S).**

**Table S1: Concentrations of redox buffer systems in the intra- or extracellular space.** References are given in parentheses.

|  | Intracellular | Extracellular (plasma) |
| --- | --- | --- |
| GSH | 1 – 10 mM [1] | 2.8 ± 0.91 µM [2] |
| GSSG | 100 µM (calculated assuming 5 mM GSH and a GSH/GSSG ratio 50:1) | 0.14 ± 0.91 µM [2] |
| Ratio GSH/GSSG | 30:1- 100:1 [1] |  |
| Cys | - | 9.7 ± 3.2 µM [2] |
| CySS | - | 40 ± 7 µM [2] |
| humanTrx | 0.1 – 2 µM [3, 4] | 1 – 5 nM (oxidized form) [3], [4] |

1. Hwang, C., A.J. Sinskey, and H.F. Lodish, *Oxidized redox state of glutathione in the endoplasmic reticulum.* Science, 1992. **257**(5076): p. 1496-502.

2. Jones, D.P., et al., *Redox state of glutathione in human plasma.* Free Radic Biol Med, 2000. **28**(4): p. 625-35.

3. Ottaviano, F.G., D.E. Handy, and J. Loscalzo, *Redox regulation in the extracellular environment.* Circ J, 2008. **72**(1): p. 1-16.

4. Nakamura, H., H. Masutani, and J. Yodoi, *Extracellular thioredoxin and thioredoxin-binding protein 2 in control of cancer.* Semin Cancer Biol, 2006. **16**(6): p. 444-51.
